# Selecting a Conservation Surrogate Species for Small Fragmented Habitats Using Ecological Niche Modelling

**DOI:** 10.3390/ani5010027

**Published:** 2015-01-05

**Authors:** K. Anne-Isola Nekaris, Andrew P. Arnell, Magdalena S. Svensson

**Affiliations:** Nocturnal Primate Research Group, Department of Social Sciences, Faculty of Humanities and Social Sciences, Oxford Brookes University, Oxford, OX3 0BP, UK; E-Mails: anekaris@brookes.ac.uk (K.A.-I.N.); andrew.arnell@yahoo.com (A.P.A.)

**Keywords:** Cinderella species, flagship, *Loris tardigradus tardigradus*, *Prionailurus viverrinus*, Sri Lanka, umbrella

## Abstract

**Simple Summary:**

Large “charismatic” animals (with widespread popular appeal) are often used as flagship species to raise awareness for conservation. Deforestation and forest fragmentation are among the main threats to biodiversity, and in many places such species are disappearing. In this paper we aim to find a suitable species among the less charismatic animal species left in the fragmented forests of South-western Sri Lanka. We selected ten candidates, using a questionnaire survey along with computer modelling of their distributions. The red slender loris and the fishing cat came out as finalists as they were both appealing to local people, and fulfilled selected ecological criteria.

**Abstract:**

Flagship species are traditionally large, charismatic animals used to rally conservation efforts. Accepted flagship definitions suggest they need only fulfil a strategic role, unlike umbrella species that are used to shelter cohabitant taxa. The criteria used to select both flagship and umbrella species may not stand up in the face of dramatic forest loss, where remaining fragments may only contain species that do not suit either set of criteria. The Cinderella species concept covers aesthetically pleasing and overlooked species that fulfil the criteria of flagships or umbrellas. Such species are also more likely to occur in fragmented habitats. We tested Cinderella criteria on mammals in the fragmented forests of the Sri Lankan Wet Zone. We selected taxa that fulfilled *both* strategic and ecological roles. We created a shortlist of ten species, and from a survey of local perceptions highlighted two finalists. We tested these for umbrella characteristics against the original shortlist, utilizing Maximum Entropy (MaxEnt) modelling, and analysed distribution overlap using ArcGIS. The criteria highlighted *Loris* *tardigradus tardigradus* and *Prionailurus viverrinus* as finalists, with the former having highest flagship potential. We suggest Cinderella species can be effective conservation surrogates especially in habitats where traditional flagship species have been extirpated.

## 1. Introduction

Considering the urgent need for conservation action surrogate species are often employed to act as conservation short-cuts, where management focused on one or a few species will benefit an ecosystem as a whole [1,2]. These surrogates may have ecological roles such as indicators and umbrellas, or strategic roles such as flagship species [3]. Flagships are single-species chosen to represent and sell the concept of maintaining biodiversity [4] to the public, and in doing so raise support for conservation [5,6,7]. The criteria for selection of flagship species are not universal, but related to the human audience that the flagship is intended to target. In addition, these traditionally large and charismatic species [8,9] are sometimes used for setting conservation priorities such as protected area (PA) selection, although their usefulness as umbrella species for regional biodiversity have been found to be limited in some areas [10,11,12].

In fragmented habitats, however, larger species and especially top predators are generally more prone to extinction [13,14,15]. Human-mediated habitat fragmentation and its associated effects are widely cited as one of the key threats to biodiversity [14,16,17,18,19,20]. If these charismatic species disappear from such areas, the outreach opportunities they bring are also lost. Consequently, smaller species may be required to act as stand-ins to the typical flagships [20,21].

Relevant to the loss of the typical charismatic species, Smith *et al.* [7] introduced the Cinderella species concept. Cinderella species may have similar profiles to flagship species, although may have previously been overlooked due to less obvious charismatic features. In response to “overrepresentation” of large charismatic species in conservation campaigns, they suggested that many species are available that would fill a similar role, and that in particular, relatively large taxa or those with forward-facing eyes might be favoured by the public. Appeal in several categories was considered important not only for selection of the surrogate, but also to appeal to donors, and such characteristics include namely sharing their range with other species, being highly sentient, being important for ecotourism, having cultural significance, or playing a role in ecological processes They also urged in the creation of a new flagship that public awareness must play a pivotal role, with the public having some type of positive feelings about the chosen species [7]. Like flagship species, the Cinderella species concept does not include as a selection criteria species with significant and beneficial ecological characteristics [5,21]. As Caro and O’Doherty ([1], p. 810) note, “Flagships need only be popular, not ecologically significant”. In areas characterised by heavy fragmentation and extirpation of most large species, selecting a flagship species appreciated by local people is especially important. The charisma of a flagship species is a selling point to the general public that may never see a Sri Lankan forest, but may respond emotionally to the thought of losing these charismatic species. The general public has little influence over the mitigation of local threats, so this is best addressed by building local support.

A case in point is the Wet Zone of Sri Lanka, a biodiversity hotspot characterised by a network of very small fragments (most less than 600 ha in size) [22,23]. Continued and unsustainable use of these fragments highly imperils their largely endemic animal populations. To improve ecotourism and conservation awareness in Sri Lanka, the Wildlife Conservation Department recently declared seven animal species as the national flagships: Asian elephant (*Elephas maximus*), sloth bear (*Melursus ursinus*), leopard (*Panthera pardus*), black-necked stork (*Ephippiorhynchus asiaticus*), saltwater crocodile (*Crocodylus porosus*), leatherback turtle (*Dermochelys coriacea*) and blue whale (*Balaenoptera musculus*) [24]. All of these taxa, however, have been virtually extirpated from the Sri Lankan Wet Zone [25]. Interest in wildlife is long-established in Sri Lanka, partially due to cultural beliefs [26]. Environmental topics have been integrated into primary and secondary curricula since the 1970s [27], with numerous eco-clubs, societies, and internet social network groups established [28,29]. This scenario makes Sri Lanka’s Wet Zone an ideal study area in which to investigate the selection of an appropriate Cinderella species.

In this study, we examined traditionally neglected mammal species occurring in Sri Lanka’s Wet Zone, selected for both their appeal to the target audience and their umbrella characteristics. Using a survey of local perceptions in combination with ecological niche modelling, we aim to select an ecologically and culturally suitable Cinderella species to act as a flagship species for Sri Lanka’s fragmented rainforest.

## 2. Experimental Section

### 2.1. The Study Area and Its Inhabitants

We restricted analysis to an approximation of the Sri Lankan Wet Zone (5.89°N–7.55°N, 79.8°E–80.95°E). Some 108 million people live in the Wet Zone at an average human population density of approximately 1000 individuals/km^2^ [30]. Of those employed (37% of the population) about 40% are listed as craftsmen or as working in an elementary occupation [30]. Rate of literacy in Sri Lanka is high at 94.2%, and internet use, including social networking sites such as Facebook (with nearly 15 million users), is also high throughout the country but especially in the heavily populated Wet Zone. Wildlife tourism in Sri Lanka is a major economic driver, with domestic tourists comprising a large proportion.

### 2.2. Shortlist Criteria and Locally Appropriate Flagship Characteristics

**Figure 1:**
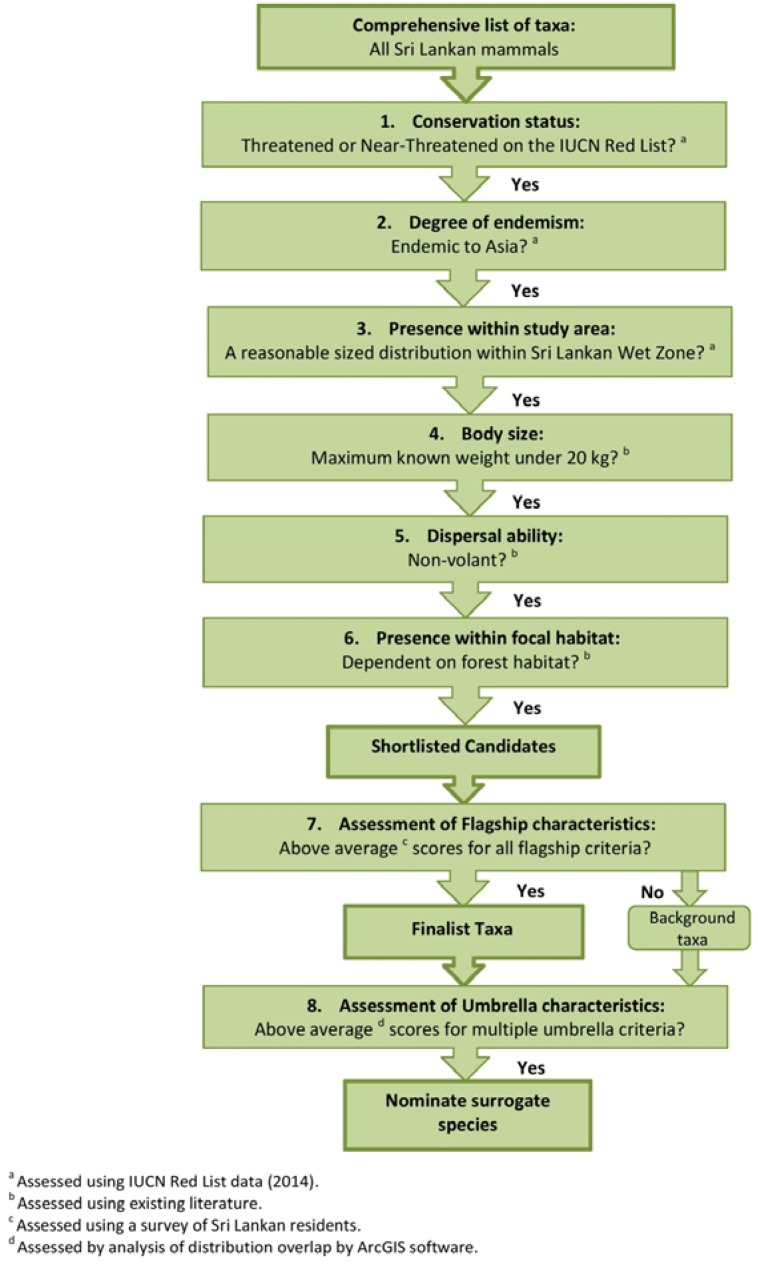
Flowchart of criteria used in the selection of a surrogate species for the Sri Lankan Wet Zone.

To compose a list of selection criteria, we adapted existing literature on flagship and umbrella species, and also followed the criteria of Smith *et al.* [7] (Figure 1). We applied these criteria to all mammals with relatively forward facing large eyes within the study area to produce a shortlist of species, referred to as candidates. We chose only non-volant mammals as they are the most likely animals to be seen in fragments without the aid of specialist techniques. We recruited participants who would potentially pay to see or support wildlife, but at a local level, from target groups of Sri Lankan residents with an interest in wildlife (*N* = 84 out of 110). Participants completed a survey that we analysed to select candidates able to fulfil the role of flagship at a local scale. Possible bias for categories could result from varying levels of species recognition, with respondents being more likely to choose taxa they had heard of previously [31]. Only 21 candidates had heard of all ten species. Thus we expressed figures for each selection criterion as a percentage of the respondents recognizing a species. This technique allowed for consideration of species known to fewer respondents; this mechanism can also serve as a tool to highlight these lesser-known preferred species in future campaigns (*cf.* [5]). To qualify as a “finalist” the candidate had to score above the mean for three criteria, each chosen to represent a flagship characteristic: (1) the respondent ranked the animal as attractive to look at; (2) the respondent considered the animal to play an ecosystem role that has easily perceivable benefits to humans (e.g., pest management, pollination, ecotourism); and (3) wildlife tourism is a thriving local industry, with life lists for bird, mammal, insect, reptile and plant species—this question thus refers to the animal’s value to wildlife enthusiasts. We used SPSS 21 for non-parametric statistical analysis with significance values set to *p* ≤ 0.05.

### 2.3. Ecological Niche Modelling (ENM)

We used ecological niche modelling software, MaxEnt [32,33] to produce distribution maps. Our chosen taxon of mammals has been well-tested with ecological niche modelling [32,34,35]. For candidates we collected locality data (1837–1979) from zoological collections (Paris, Washington, Chicago, London, Oxford, Amsterdam, Leiden) to represent their historic range for the purposes of modelling, eight years of field data collected by Nekaris and colleagues at 47 localities in the Wet Zone, communication with researchers and available literature. Where necessary, we geo-referenced localities using Google Earth.

We used the most recent land cover layer available [36] and produced habitat layers for each candidate. We excluded species localities over 2 km outside their habitat layer, as well as peripheral localities dating prior to 1970. To achieve independence of data we only used localities more than 10 km apart [35,37].

We utilized the ENM software, MaxEnt for the modelling process, as it is able to work with presence only data and can be effective with as few as five localities [37]. From a total of 249 collected localities we used 30% for testing the models and 70% for training, as this proportion gave the highest area under the curve (AUC) values [32,37,38]. We entered the locality data into MaxEnt, along with 20 environmental layers [39] at ~1 km^2^ resolution. To distinguish presence from absence, we chose a fixed threshold of 10% as it allowed for a reasonable level of omission [37]. We projected resultant maps in ArcGIS 10.1 where we clipped species distributions by their corresponding habitat layers and excluded fragments under 5 km^2^, as this was regarded as the minimum viable fragment size for the study species [40,41].

### 2.4. Assessment of Umbrella Characteristics

From the modelled distributions, we carried out a comparison of spatial overlap between all ten species (*cf.* [42]). We focused our results on the finalists, but for comparison produced results for all short-listed candidates. We employed three criteria for assessing umbrella characteristics:

(1) Overlap in geographic range: For each species acting as umbrella, we calculated the percentage of the beneficiary species’ range in the study area that occurred within the range of the umbrella [42].
(2) Number of species protected: If more than 75% of the beneficiary species’ range within the study area was afforded cover, we counted it as “protected”.
(3) Umbrella efficiency was defined as the level of overlap per km^2^ of the umbrella’s range. We calculated this for each umbrella by dividing the mean area of overlap from all beneficiary species, by the umbrella’s total range within the study area.

We used the first two criteria to find a species with effective umbrella characteristics for the study area as a whole. We included a third criterion, umbrella efficiency, to take into account the size of the umbrella species’ distribution. We named the finalist with the highest scores for multiple umbrella criteria as the Cinderella species for the study area. The nominate Cinderella species had to score higher for all umbrella criteria than a random distribution of equal area [43,44].

## 3. Results

### 3.1. Shortlist Criteria and Locally Appropriate Flagship Characteristics

The IUCN Red List [25] lists 92 terrestrial mammals in Sri Lanka. Using the Cinderella criteria, we successfully reduced this to a shortlist of ten candidates of all non-volant mammals [45] and known to occur in the lowland Wet Zone tropical rainforests of Sri Lanka. All were listed in the IUCN Red List [25] as Near-Threatened, Threatened, Endangered, or Critically Endangered. The one exception was the Least Concern yellow-spotted mouse deer (*Moschiola kathygre*), included as it is known to be suffering sharp declines in the study region [46], and because Smith *et al.* [7] state that conservation status is not necessarily key to Cinderella effectiveness.

**Figure 2:**
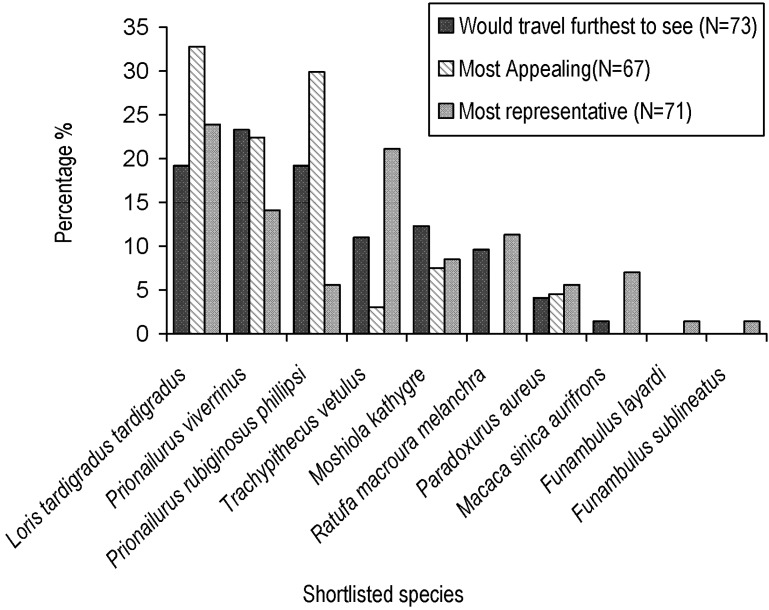
A comparison of three flagship characteristics for shortlisted conservation surrogate candidates.

Eighty four Sri Lankan residents completed questionnaires, out of 110 potential respondents that were contacted. From these data we highlighted two species as finalists, red slender loris (*Loris tardigradus tardigradus*) and fishing cat (*Prionailurus viverrinus*), as these were the only species with above average (>10%) scores for all three flagship criteria (Figure 2). Responses were significant for each of the three criteria assessed: “appeal” (χ^2^ = 48.23, df = 9, *p* < 0.01), “rainforest representation” (χ^2^ = 37.873, df = 9, *p* < 0.01), and “would travel furthest to see” (χ^2^ = 104.19, df = 9, *p* < 0.01). The rusty-spotted cat (*Prionailurus rubiginosus phillipsi*) and southern purple-faced langur (*Trachypithecus vetulus vetulus*) scored above average but not for all three categories. The lowest scoring species were Layard’s palm squirrel (*Funambulus layardi*) and the dusky striped squirrel (*F. sublineatus*). Comparing scores against group averages, after controlling for recognition, produced the same trends, with *L. t. tardigradus* and *P. viverrinus* both having greatest potential flagship characteristics.

### 3.2. Ecological Niche Modelling

**Figure 3:**
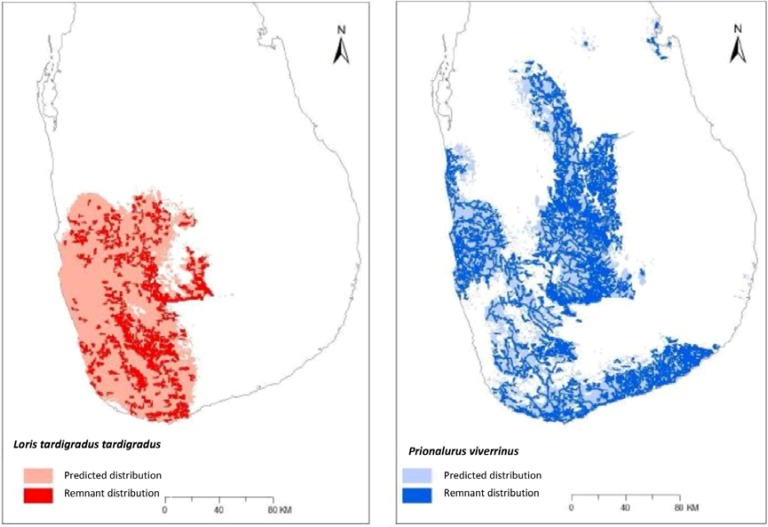
Maxent-generated predicted and remnant distributions of *Loris tardigradus tardigradus* and *Prionailurus viverrinus*, the two taxa with the highest overlap efficiency.

According to their AUC curves, all ten models performed better than random (>0.50). The Sri Lankan giant squirrel (*Ratufa macroura melanochra*) scored highest (0.96); whereas *P. viverrinus* performed least favourably (0.70). The distribution of each candidate within the study area varied widely *P. viverrinus* and *M. kathygre* had the highest distribution overall, each covering 34% of the study area, *L. t. tardigradus* covered approximately half this and the lowest distribution was *T. v. vetulus*, covering only 7.7% of the area (Figure 3).

### 3.3. Overlap within Geographic Range

*Loris t. tardigradus* had a higher overlap within geographic range (68.8%) than *P. viverrinus* (56.8%). *Loris t. tardigradus* also scored higher than the group average for this category. When considering all candidates, the highest overlap within range was *M. kathygre* (86.6%); *T. v. vetulus* was lowest (41.6%).

### 3.4. Number of Species Protected

Protecting the whole of the range of *L. t. tardigradus* would afford at least 75% cover to five of the nine beneficiary species, whereas *P. viverrinus* did not afford protection to any species at this level of cover. When the cover threshold was increased to 95% the number protected by all candidates dropped significantly and only *M. kathygre* was able to protect a reasonable number of species (Table 1). This threshold is an unlikely conservation goal in practice. Despite having the largest distribution, *P. viverrinus*, was only effective at the lowest threshold of cover (50%).

**Table 1:** Comparison of effectiveness of Sri Lankan Wet Zone mammal species at affording umbrella protection at three different thresholds of cover.

Candidates	Protection Afforded to Beneficiary Species		
	No. of Species Afforded >50% Cover	No. of Species Afforded >75% Cover	No. of Species Afforded >95% Cover
*Prionailurus rubiginosus phillipsi*	8	7	2
*Moschiola kathygre*	9	9	3
*Ratufa macroura melanochra*	4	1	1
*Prionalurus viverrinus*	6	0	0
*Paradoxurus aureus*	7	3	0
*Funambulus layardi*	3	0	0
*Funambulus sublineatus*	6	2	0
*Trachypithecus vetulus vetulus*	2	0	0
*Macaca sinica aurifrons*	7	4	0
*Loris tardigradus tardigradus*	7	5	1

### 3.5. Overlap Efficiency

From the two finalists *L. t. tardigradus* had the highest efficiency (61.5%) and *P. viverrinus* had the lowest of any taxon (27.7%). The mean overlap efficiency of all study species was 62.3%, and neither finalist scored above this. Among all candidates, *T. v. vetulus* had the highest efficiency (85.5%). We found the scores for overlap efficiency to be negatively correlated with the number of different habitat types used for modelling each species (r_s_= −0.753, df = 8, *p* < 005). This association suggests that within this study, specialist species seem to exhibit stronger overlap efficiency.

### 3.6. Selecting Cinderella Species

Results between criteria varied widely with certain species, most notably those with large or small geographic range, such as *T. v. vetulus* and *M. kathygre* (Figure 2). From the finalists, *L. t. tardigradus* fared well for all three criteria although scored slightly below average for overlap efficiency, whereas *P. viverrinus* scored below average for all umbrella criteria. Using all three umbrella criteria, *L. t. tardigradus* was the highest scoring finalist and can be classed as the most appropriate Cinderella species.

## 4. Discussion

Combining local opinion with ecological niche modelling on mammals in Sri Lanka’s Wet Zone highlighted *L. t. tardigradus* as the most appropriate surrogate species candidate. From the results of the survey, both finalists, *P. viverrinus* and *L. t. tardigradus*, were well-regarded. Following Smith *et al.* [7] both candidates exhibited the typically favoured characteristic of forward-facing eyes, and fell amongst the most popular flagship Orders (Primates and Carnivora). *Loris t. tardigradus*, however, achieved higher scores for all three umbrella criteria and was selected as a more ideal surrogate species for this highly fragmented forest network.

Considering primates are revered in many cultures, including in Sri Lanka [26,47] and are already commonly used as flagships in other locations [4,5], *L. t. tardigradus* appears a plausible choice of flagship species. Kinan and Dalzell [48] note, however, that the use of species as flagships might exacerbate already existing conflicts. They found that using sea turtles as flagships in the Pacific Islands actually created more conflict between NGOs and local residents. Historically slender lorises were used for various traditional practices both medical and ethnographical, although such practices are now considered very rare [49]. Indeed, Nekaris *et al.* [49] showed that lorises may also be considered to have an important spiritual role to some local cultures within Sri Lanka, improving its utility as a conservation surrogate. Furthermore, in several recent “primate-human conflict” surveys, *Loris* was considered by local people to be a shy and innocent animal [50]. *Loris* is also the chosen name for the journal of the Wildlife and Nature Protection Society of Sri Lanka, originally designated as such as it was considered to be “redolent of the mystic aspects of the jungle” of Ceylon [51]. This species’ umbrella characteristics should, if its habitat is protected, confer >75% cover to five of the nine beneficiary species. A non-saltatory arboreal specialist, *L. t. tardigradus* is confined to areas of contiguous forest [41], meaning protecting it would also drive the preservation of the most intact of the Wet Zone’s remaining forests.

From a shortlist of ten Cinderella species, only *L. t. tardigradus* and *P. viverrinus* appeared likely to fulfil a local flagship role. Our findings echo previous studies [52] that primates and felids are generally the most appealing taxa. The associations that some primate species have with crop raiding, such as the dusky toque monkey (*Macaca sinica aurifrons*) and *T. v. vetulus*, [53], may explain their lower scores relative to *L. t. tardigradus*, which has no such association [26,47]. The ENM process produced models that performed better than average for all species, despite a small number of localities available for some taxa (<20), reiterating that MaxEnt can be effective with as low as five localities [37]. Only one model, for *P. viverrinus*, had a relatively low AUC value, however this case is not likely to suggest a poor model, but more a reflection of the species’ widespread distribution throughout Sri Lanka [35,38].

Our aim was to select for multiple criteria and therefore choose a trade-off between conservation goals and to appeal to multiple stakeholders [54,55]. In our study *T. v. vetulus* had highest scores for efficiency but a limited distribution (7.7% of area) and so performed poorly for criteria assessing the study area as a whole. This disparate performance re-iterates that high performance in one umbrella characteristic may not be indicative of overall umbrella utility [43]. The significant negative correlation between habitat preferences and overlap efficiency suggests that the specialist species appear to have higher overlap efficiency. Cabeza [56] and Suter [57] both agree that generalists may not make particularly strong umbrellas, despite often covering a larger geographic range. A degree of caution should always be involved in generalizing from umbrella studies [43]. Currently little standardization exists for the assessment of umbrella species [2,43,44,58] and we suggest that a set umbrella methodology, such as detailed in this study, be standard. We also suggest that conservationists can use data obtained from modelling to put forward new taxa that may initially score lower in appeal. For example, conservation education programmes can use a species such as *L. tardigradus tardigradus* as a gateway to introduce local people to the positives and unusual aspects of animals that could afford greater cover but scored lower in flagship characteristics.

We chose to incorporate local people who would be likely eco-tourists into our methodology, increasing the likelihood of strong local support for the nominated species [59]. At the village level, education initiatives may benefit from emphasizing ecological benefits brought by species that people can see as directly impacting their own lives (e.g., pollination, pest control) and improve public acceptance of formation of corridors [21,60]. Managers of such systems can also use data collected from such studies to help bridge the gap between attraction to more typically charismatic species and the need to preserve the integrity of the ecosystem, again with positively received Cinderella species as pathways to introducing more atypical taxa [61].

With the world’s forests disappearing at an alarming rate, conservation of fragmented habitat becomes essential. In such habitats, people are more likely to live alongside potential surrogate species, thus making the role of people in the selection of such species even more important. The process used here to select a surrogate species for fragmented habitat was efficient and based on a set of scientific criteria. We feel that a combination of interviews, ground truthing and GIS modelling can easily be adapted in other habitats to choose a species that can form the role of multiple surrogate concepts.
